# Comprehensive Assessment of Antioxidant and Anti-Inflammatory Properties of Papaya Extracts

**DOI:** 10.3390/foods11203211

**Published:** 2022-10-14

**Authors:** Yoon A Jeon, Sun Woo Chung, Seong Cheol Kim, Young Jae Lee

**Affiliations:** 1 College of Veterinary Medicine, Jeju National University, Jeju 63243, Korea; 2 Research Institute of Climate Change and Agriculture, National Institute of Horticultural and Herbal Science, Jeju 63240, Korea

**Keywords:** ABTS, FRAP, flavonoid, NF-κB, phenolic, ripening, seed

## Abstract

Antioxidant and anti-inflammatory properties of papaya (*Carica papaya*) fruits were evaluated to provide comprehensive information associated with the bioactive compounds. ‘Tainung No. 2’ papaya fruits, cultivated in a greenhouse, Korea, were harvested at unripe and ripe stages and then divided into seed and peel-pulp. Total phenolic and flavonoid contents were determined using spectrophotometry, and individual phenolic compounds were relatively quantified by HPLC-DAD and fifteen standards. Antioxidant activities were measured using four assays: DPPH (2,2-diphenyl-1-picrylhydrazyl) and ABTS (2,2′-azino-bis (3-ethylbenzothiazoline-6-sulfonic acid)) scavenging activities, inhibition of lipid peroxidation, and FRAP (ferric reducing antioxidant power). Anti-inflammatory activities were measured by the regulation of NF-κB signaling pathways with the measurements of ROS and NO productions as the degree of oxidative stress. Total phenol contents increased in seed and peel–pulp extracts during ripening; flavonoid contents increased only in seed extracts. Total phenolic contents were associated with ABTS radical scavenging activity and FRAP. Of fifteen phenolic compounds, chlorogenic acid, cynarin, eupatorine, neochlorogenic acid, and vicenin II were identified among papaya extracts. ROS and NO productions were inhibited in papaya extracts. Especially, NO productions were inhibited higher in ripe seed extracts than in other extracts, which would be associated with the suppression of NF-κB activation and iNOS expression. These results suggest that papaya fruit extracts, including seeds, peels, and pulps, could be potential raw materials for functional foods.

## 1. Introduction

Papaya (*Carica papaya*) belongs to the Caricaceae family and originates from Mexico and South America [1]. It is a globally popular subtropical/tropical fruit crop used for food and medicine owing to its nutritional and phytochemical composition [2,3]. Papaya fruit is traditionally consumed in its unripe and ripe forms, which have distinct tastes, aromas, and nutrient content. The fruit is consumed fresh or processed as part of salads, desserts, and beverages, among other foods. Papaya cultivation has expanded from subtropical and tropical regions to temperate regions, including Japan and Korea. In these regions, greenhouse cultivation is essential because low temperatures in the winter season can damage subtropical and tropical crops, including avocado [4], coffee [5], papaya [6], and mango [7]. Greenhouse cultivation can affect fruit characteristics through artificial climatic conditions, including solar radiation, temperature, and irrigation [8]. However, there is still little information on the qualities of papaya fruits cultivated in a greenhouse, despite the rapid expansion of the cultivation in temperate regions.

Papaya fruit accumulates various bioactive compounds, including phenolics, carotenoids, saponins/triterpenoids, and ascorbic acid, which have pharmacological effects [1]. Phenolics are the most important bioactive compounds in papaya fruits [9]. Phenolics include phenolic acids, flavonoids, stilbenes, and lignans, which differ based on the number of phenol rings and the structural components that bind these rings [10]. Flavonoids are the main group of polyphenols, with diverse subgroups, including anthocyanins, flavonols, and tannins. Papaya fruit accumulates phenolic acid [11], lignan [12], and flavonoids [13,14]. The main flavonoid compounds in the papaya peels, pulps, and leaves were determined to be myricetin, quercetin, kaempferol, morin, apigenin, and luteolin. The contents and compositions of phenolics vary at ripening stages and parts of papaya fruit [9].

Phenolics have various bioactive properties and prevent cells from unfavorable conditions. Exposure to external stimuli induces oxidative stress in human bodies through the overproduction of reactive oxygen species (ROS) generated in various sources, including mitochondrial respiratory chains, cytochrome p450, and NADPH oxidases [15]. Oxidative stress contributes to the onset and/or progression of several diseases, including cancers, metabolic disorders, and cardiovascular diseases [15]. Under excessive ROS formation, phenolics can directly or indirectly exert defense mechanisms. The ROS-scavenging activities of phenolics are structurally attributed to the presence of a benzene ring-bound hydroxyl that can react with ROS, including hydroxyl, superoxide, and peroxyl radicals [16]. Phenolics also exhibit anti-inflammatory activities by regulation of signaling pathways, including NF-κB and MAPKs [17,18]. NF-κB, a transcription factor, is a transducer of extracellular stress stimuli to intracellular responses, which is associated with the production of various pro-inflammatory mediators, including inducible nitric oxide synthase (iNOS), cyclooxygenase-2 (COX-2), and cytokines [19,20]. iNOS is a macrophage-type enzyme that can produce excessive NO by stimulating oxidative stress and pro-inflammatory signals [21]. The accumulation of excessive NO is associated with human diseases, including cancer and inflammation [22]. COX-2 is one of the key mediators of inflammatory signaling, and the increase in the expression is associated with various cancers and inflammation [23].

The evaluations of various antioxidant and anti-inflammatory properties are complex, especially in crops, due to physiological parameters, including different tissues and the development stages [9]. Standard techniques for the evaluation were not well-developed due to numerous bioactive compounds and their complicated interactions. Therefore, extensive studies are continuously required to obtain a more comprehensive view of these activities. In this study, papaya fruits were cultivated and collected in Korea, a newly developed area for papaya cultivation. The phenol contents were determined in different parts of unripe and ripe papaya fruits. The comprehensive antioxidant and anti-inflammatory activities were profiled in each part of papaya fruits during ripening with the inhibitory effects of ROS and NO production. Antioxidant and anti-inflammatory properties were measured in terms of chemical, structural, and biological aspects. This study provides a conceptual framework for the further study of papaya antioxidant properties.

## 2. Materials and Methods

### 2.1. Sample Preparation

‘Tainung No. 2’ papaya trees were planted in November 2015 at a greenhouse at the experimental orchard of the Research Institute of Climate Change and Agriculture, the National Institute of Horticultural and Herbal Science, Jeju, Korea (33°28′ N, 126°31′ E). The papayas were cultivated under a standard guideline for papaya cultivation [24]. The minimum temperatures of the winter season were maintained above 15 °C to prevent papaya trees from chilling or freezing injuries [24]. According to Addai, Abdullah and Mutalib [13], fruit were categorized into two ripening stage based on weeks after fruiting (WAF): unripe at ca. 12 WAF and ripe at ca. 20 WAF. In June 2021, fifteen fruits at each ripening stage were harvested from three trees to provide three replicates with five fruit each in the sampling design. The samples were immediately separated into seed and peel-pulp. The samples were dried at 25 °C and ground using a mortar and pestle. For extraction, the ground samples with 70% methanol solution were sonicated two times in an ultrasonic bath (JAC-3010, Kodo Technical Research Co., Ltd., Hwaseong, Korea) at 25 kHz frequency for 30 min at 25 °C. The homogenates were filtered using a filter paper (F1001, Chimlab group, Barcelona, Spain) and evaporated in a rotary evaporator (IKA RV8, IKA-Werke GmbH & Co. KG, Staufen, Germany) at 50 °C. The extracts were freeze-dried and stored at −20 °C before further analyses.

### 2.2. Determination of Phenol Contents

#### 2.2.1. Total Phenol and Flavonoid Contents

Total phenolic contents were determined using the Folin-Deni method [25]. The papaya extracts were reacted with Folin–Ciocalteu’s phenol reagent and a solution of 7% Na_2_CO_3_ (*w*/*v*) for 60 min at ambient temperature. The absorbance was measured at 720 nm using a microplate reader (SpectraMax M3, Molecular Device, San Jose, CA, USA). The total phenolic content was expressed as mg gallic acid equivalents per 100 g of dry weight (mg GAE/100 g DW) 

Total flavonoid contents were determined as previously described in Quettier-Deleu et al. [26]. In brief, the extracts were reacted with a solution of 2% AlCl_3_ (*v/v*) for 15 min at ambient temperature. The absorbances were measured at 430 nm using a microplate reader (SpectraMax M3). The total flavonoid content was expressed as mg quercetin equivalents per 100 g of DW (mg QUE/g DW).

#### 2.2.2. Individual Phenolic Contents

Papaya extracts were separated in a Hydro-RP C-18 column (250.0 mm × 4.6 mm, 5 µm, Phenomenex, Torrance, CA, USA) equipped with HPLC system (1260 Infinity II LC system, Agilent, Santa Clara, CA, USA) fitted with a diode array detector set at 340 nm. Eluents were passed through the column at a flow rate of 1 mL/min using a gradient of solvent A (water) and solvent B (acetonitrile) in the following sequence: 0–5 min, 2% B; 5–12 min, 2–5% B; 12–17 min, 5–8%; 17–65 min, 8–30% B; 65–68 min, 30% B; 68–78 min, 30–50% B; 78–100 min, and 50–100% B. For the quantification of phenolics, relative retention times and relative peak areas were compared using fifteen compounds as standards: acacetin (CAS 480-44-4), apigenin (CAS 520-36-5), apigetrin (CAS 528-74-5), chlorogenic acid (CAS 327-97-9), cryptochlorogenic acid (CAS 905-99-7), cynarin (CAS 30964-13-7), diosmin (CAS 520-27-4), eridictol (CAS 20126-59-4), eupatorine (CAS 855-96-9), isoschaftoside (CAS 52012-29-0), linarin (CAS 480-36-4), luteolin (CAS 491-70-3), luteoside (CAS 5373-11-5), neochlorogenic acid (CAS 906-33-2), and vicenin II (CAS 23666-13-9).

### 2.3. Determination of Antioxidant Activities

#### 2.3.1. DPPH Radical Scavenging Activities

DPPH radical scavenging activities were evaluated following a previously described in Blois [27] with some modifications. The samples were reacted with 0.2 mM DPPH for 10 min at ambient temperature, and the absorbances were measured at 517 nm using a microplate reader (SpectraMax M3). DPPH radical scavenging activities were calculated with Equation (1).

(1) $$\mathrm{Radical}\;\mathrm{scavenging}\;\mathrm{activity}\;\left(\%\right)=1\;-\;\frac{\mathrm{Absorbance}\;\mathrm{of}\;\mathrm{sample}}{\mathrm{Absorbance}\;\mathrm{of}\;\mathrm{control}}\times 100$$

The concentrations required to obtain a 50% antioxidant effect (EC_50_) were determined for DPPH radical scavenging activities. According to [28], Equations (2) and (3) were implemented for non-linear regression.

(2) $$Y=\frac{1}{1+10^{\left\{\left[\log{\mathrm{EC}}_{50\;}-\log x\right]\;\times \;\mathrm{Hillslope}\right\}}}$$

(3) $$Y=\;\mathrm{Bottom}\;+\frac{\mathrm{Top}\;-\;\mathrm{Bottom}}{{\left(1+10^{\left[\left(\log x_{b}-\log x\right)\times \mathrm{Hillslope}\right]}\right)}^{s}}$$

#### 2.3.2. ABTS Radical Scavenging Activities

ABTS radical scavenging activities were evaluated according to Re et al. [29]. A solution of 7.4 mM ABTS was mixed with 2.6 mM potassium persulfate for 16 h, which converted ABTS to ABTS^+^. The samples were reacted with the diluted solution of the ABTS^+^ for 15 min, and their absorbances were measured at 734 nm using a microplate reader (SpectraMax M3). ABTS radical scavenging activities and EC_50_ were calculated using the same equation to determine the DPPH radical scavenging activities.

#### 2.3.3. Lipid Peroxidation (LPO) Inhibition

The inhibitions of LPO were determined using 2-thiobarbituric acid reactive substances assay according to Ahn et al. [30]. The livers of Wistar-Tokyo rats were homogenized in 50 mM sodium phosphate buffer (pH 7.4), and protein contents were quantified using the Bradford method [31]. First, 0.5 mg of the protein was reacted with a solution of 0.1 mM FeSO_4_ and 1 mM ascorbic acid at 37 °C for 30 min. Then, the mixtures were incubated with 1% thiobarbituric acid and trichloroacetic acid at 37 °C for 5 min and then centrifuged for 15 min at 2500× *g* and 4 °C. Absorbances were measured at 532 nm using a microplate reader (SpectraMax M3). The inhibitions of LPO and the EC_50_ were calculated using the same equation to determine the DPPH radical scavenging activities.

#### 2.3.4. FRAP

Papaya extracts were reacted with 300 mM acetate buffer, 10 mM 2,4,6-tripyridyl-s-triazine, 40 mM HCl, and 20 mM FeCl_3_·6H_2_O for 4 min at 37 °C, according to Benzie and Strain [32]. The absorbances were measured at 593 nm using a microplate reader (SpectraMax M3). FRAP was expressed as µg FeSO_4_·7H_2_O equivalents per gram of DW (µg FeSO_4_·7H_2_O/g DW).

### 2.4. Determination of Anti-Inflammatory Activities

#### 2.4.1. Cell Culture and Sample Preparation

RAW-Blue™ cells (InvivoGen, San Diego, CA, USA) were propagated in Dulbecco’s modified Eagle’s medium (Gibco, Carlsbad, CA, USA) supplemented with 10% fetal bovine serum (Gibco) and 1% penicillin/streptomycin at 37 °C and 5% CO_2_. The cells were seeded in 96-well plates at a density of 1 × 10^5^ cells/mL and then incubated with 200 μg/mL of the papaya extracts for 1 h. Lipopolysaccharide (LPS; 1 μg/mL) was then added for 24 h prior to further analyses.

#### 2.4.2. Cell Viabilities

The viabilities of RAW-Blue™ cells were assessed using MTT (3-(4,5-dimethylthiazol-2-yl)-2,5-diphenyltetrazolium bromide) tetrazolium reduction assay [33] to determine the cytotoxicity of papaya fruit extracts. The cells in 96-well plates were incubated with the MTT solution for 4 h, and the supernatant was then obtained. The incubated cells were decrystallized by adding 200 μL dimethyl sulfoxide to each well. Absorbances were measured at 540 nm using a microplate reader (SpectraMax M3). Cell viabilities were calculated relative to the values of control wells and reported as the percentage of cell viabilities.

#### 2.4.3. Measurement of ROS Production

ROS production was measured in the RAW-Blue™ cells using fluorescence microscopy, according to Martínez and Durantini [34]. H_2_DCFDA (Invitrogen, Eugene, OR, USA) as a fluorescent probe was added to each plate well, and the cells were incubated further for 30 min at room temperature. Images of stained cells were obtained using a fluorescence microscope (CKX41, Olympus, Tokyo, Japan). The fluorescence intensity of the images was calculated using ImageJ software version 1.8.0 [35].

#### 2.4.4. Measurement of NO Production

NO production was measured in RAW-Blue™ cells through the analysis of nitrite levels using the Griess assay [36]. After the Griess reagent was added to the cells, the absorbance was measured at 540 nm using a microplate reader (SpectraMax M3). The inhibition of NO production was calculated relative to values of the LPS-treated control and was reported as a percentage of NO production. 

#### 2.4.5. Measurement of NF-κB Activation

According to Liao et al. [37], the inhibitions of NF-κB activation were measured in the supernatants of RAW-Blue™ cells mixed with QUANTI-Blue™ (InvivoGen) reagent at 37 °C for 1 h. The absorbances were then measured at 620 nm. The inhibitions of NF-κB activation were calculated relative to values of the LPS-treated control and reported as the percentage of secreted embryonic alkaline phosphate.

#### 2.4.6. Measurement for iNOS and COX-2 Expressions

The RAW-Blue™ cells were washed with phosphate-buffered saline and then lysed in RIPA lysis buffers on ice for 30 min. After centrifugation at 10,000× *g* for 10 min at 4 °C, the protein contents of the cells were determined by the Bradford method [31]. The 30 µg of protein samples were separated with SDS gel electrophoresis and transferred onto polyvinylidene fluoride membranes. The membranes were blocked with 5% blocking grade buffer in TTBS (tris-buffered saline containing 0.1% Tween-20) for 1 h, and then incubated with mouse monoclonal antibodies against iNOS, COX-2, and β-actin (1:1000 dilution; Santa Cruz Biotechnology Inc., Santa Cruz, CA, USA). Finally, the membranes were incubated with HRP-linked secondary antibodies (1:2000, mouse IgG antibody-HRP; GeneTex, CA, USA) for 40 min at 25 °C. The membranes were washed with TTBS after each antibody-binding reaction. Each protein was detected using a Western blot imaging system (Fusion Solo S Chemi-Doc, Vilber, Marne-la-Vallée, France) system with an electrochemiluminescence kit (Amersham Biotech, Little Chalfont, UK) according to the manufacturer’s instructions (Amersham).

### 2.5. Statistical Analyses

All statistical analyses were performed using GraphPad Prism version 6.0.0 for Windows (GraphPad Software, San Diego, CA, USA). Statistically significant differences were determined by one-way ANOVA. Means were compared using Tukey’s honestly significant difference test at *p* < 0.05.

## 3. Results and Discussion

### 3.1. Total Phenol and Flavonoid Contents

Phenol contents differed significantly among papaya extracts, ranging from 235 to 2070 mg GAE/100 g DW (Table 1). At all ripening stages, the phenolic contents in seeds were higher than those in the peel–pulps. The differences in phenol contents between the peel-pulps and seeds decreased between two- and nine-folds during ripening. The contents in the seeds decreased from 2070 to 1080 mg GAE/100 g DW, whereas that in the peel-pulps increased from 235 to 568 mg GAE/100 g DW during ripening.

Total flavonoid contents ranged from 61.9 to 117.7 mg QUE/100 g DW among papaya extracts (Table 1). During the ripening of papaya fruits, the contents in the seeds changed from 61.9 to 117.7 mg QUE/100 g DW, whereas that in the peel-pulps were maintained at 69.75 mg QUE/100 g DW. The differences in flavonoid contents between the seeds and peel-pulps were not significant at the unripe stage but were significant at the ripe stage.

Under unfavorable conditions, various metabolic processes within cells can generate excessive ROS, causing oxidative stress, which damages tissue and drives pathogenesis as well as aging [38]. Antioxidant compounds found in fruit can counteract such oxidative stress. In papaya seed, total phenolic content ranged from 21.93 to 77.91 mg GAE/100 g DW[3,39]. In papaya pulp, values ranged from 0.02 to 75.7 mg GAE/100 g fresh weight and up to 1263 mg GAE/100 g DW [2,40]. The increasing tendency of total phenol contents during ripening has been shown in papaya [13] as well as other fruits, including apple [41], blueberry [42,43], and grape [44]. The accumulation pattern during fruit ripening would be associated with transcriptional changes in the shikimic acid and phenylpropanoid pathways [41,43]. However, there is little transcriptional information for characterizing papaya fruit during ripening. Therefore, further study is essential to understand the accumulation pattern of phenolics in papaya fruits during ripening. In this study, the total phenolic content in seeds was relatively high, with flavonoids accumulating in the late ripening stage. Such accumulation is generally observed in many plants and is attributed to seed protection from abiotic and biotic stresses during development and germination [45]. Therefore, the papaya seed may provide raw material for natural antioxidants, in addition to papaya peel and pulp, which are already used commercially.

### 3.2. Individual Phenol Contents

Three phenolic acids (chlorogenic acid, cynarin, and neochlorogenic acid) and two flavonoids (eupatorine and vicenin II) were identified among papaya extracts (Table 2), compared to the relative retention time of fifteen phenolic compounds as standards (Figure S1). Of three phenolic acids, chlorogenic acid was identified only in unripe peel–pulp extracts. Cynarin was identified in all the peel–pulp extracts, and the contents decreased during ripening. Neochlorogenic acid was found in the seed extracts as well as the peel–pulp extracts; the contents were the highest (8.73 ± 0.16) in unripe seed extracts and the lowest (0.90 ± 0.06) in ripe seed extracts. The contents of neochlorogenic acid did not change in peel–pulp extracts during ripening; that decreased to about nine times in seed extracts. Of flavonoids, eupatorine was identified in seed extracts during ripening, and the content decreased from 4.70 ± 0.43 to 2.02 ± 0.11. Vicenin II was identified in ripe peel–pulp and seed extracts.

Although total phenolic and flavonoid contents in papaya fruits were continuously reported previously [2,3], there is little information about individual phenolic compounds in papaya fruits. The ripe pulps of ‘Sel-42’ and ‘Tainung’ papaya had twelve phenolic compounds, including caffeic acid, ferulic acid, *p*-coumaric acid, chlorogenic acid, myricetin, and *p*-hydroxybenzoic acid [46]. Eleven and five phenolic compounds were identified in the peels and pulps of ’Maradol’ papaya fruits, respectively, including caffeic acid and ferulic acid. Gayosso-García Sancho et al. [47] reported three phenolic acids (caffeic acid, *p*-coumaric acid, and ferulic acid) in the ripe peel of ‘Maradol’ papaya. In ‘Formosa’ papaya fruit, five and two phenolic compounds were identified in peel and pulp, respectively [48]. In the seed of papaya fruits, twenty-three phenolic compounds were identified [49]. The identification varied depending on cultivars, growing conditions, and extraction techniques [11]. In this study, five phenolic compounds were identified. Although chlorogenic acid was already identified in papaya fruits [46], the other four compounds were not reported yet. Of these compounds, neochlorogenic acid is an isomer of chlorogenic acid and can exert neuroprotective effects by reducing neuroinflammation and preventing rheumatoid arthritis, which was associated with the regulations of AMPK/Nrf pathways [50,51]. Cynarin, eupatorine, and vicenin II were accumulated at specific tissue and ripening stage. These accumulation patterns were shown in other phenolic compounds. However, the metabolism associated with the accumulation was not fully understood due to the complication of phenylpropanoid biosynthesis pathway. The identification of phenolic compounds would contribute to the expansion of the use of papaya extracts.

### 3.3. Antioxidant Activities

**Figure 1:**
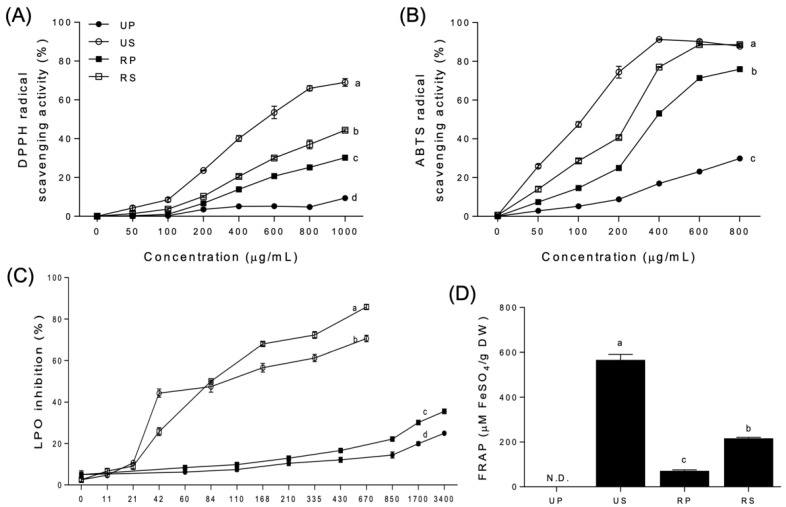
Antioxidant activities of papaya fruit extracts. (**A**) 2,2′-diphenyl-1-picrylhydrazyl (DPPH) radical scavenging activity. (**B**) 2,2′-azino-bis-(3-ethylbenzothiazoline-6-sulfonate) (ABTS) radical scavenging activity. (**C**) lipid peroxidation (LPO) inhibition. (**D**) ferric ion reducing ability of plasma (FRAP). UP, unripe peel-pulp; US, unripe seed; RP, ripe peel-pulp; RS, ripe seed. Different letters indicate significant differences using the Tukey’s honestly significant difference test at *p* < 0.05. Vertical bars represent the standard errors of means. (n = 3).

The antioxidant activities of papaya extracts varied depending on the oxidizing agents, ripening degrees, and fruit parts (Figure 1). All seed extracts exhibited higher antioxidant activities than their peel-pulp counterparts above a specific concentration, based on DPPH and ABTS radical scavenging activity as well as LPO inhibition (Figure 1A–C); FRAP was also higher for the seed extracts than for the peel-pulp extracts (Figure 1D).

DPPH radical scavenging activities were highest for the unripe seed extracts, followed by the ripe seed, ripe peel–pulp, and unripe peel–pulp extracts (Figure 1A). The order was maintained at all concentrations tested, with activities increasing up to 69.02, 44.36, 30.24, and 9.41%, respectively, at 1000 µg/mL. In the regression analysis, the activities of unripe seed, ripe seed, and ripe peel-pulp extracts showed sigmoid curves as the concentrations. However, the EC_50_ was not significantly different among the papaya extracts. The unripe peel-pulp extracts were not fitted in the regression, and the EC_50_ was not determined.

The ABTS radical scavenging activities (Figure 1B) were similar to DPPH radical scavenging activities (Figure 1A). Ripe seed extracts were the most potent, from 50 µg/mL to 400 µg/mL, followed by unripe seed, ripe peel-pulp, and unripe peel-pulp extracts. At 600 and 800 µg/mL, the antioxidant activities of unripe and ripe seed extracts were the highest, followed by those of ripe and unripe peel-pulp extracts. In the regression analysis, the ABTS radical scavenging activities of all papaya extracts, except those of the unripe peel-pulp extracts, were fitted with sigmoid curves. The EC_50_ of unripe seed extracts (119.9 µg/mL) was the lowest, followed by that of ripe seed (256.2 µg/mL) and ripe peel–pulp (349.9 µg/mL) extracts. The EC_50_ of the unripe peel–pulp extracts was not determined, exhibiting a very wide 95% confidence interval.

The LPO inhibition increased significantly for unripe and ripe seed extracts than for the peel-pulp extracts at 21 µg/mL and above (Figure 1C). Regardless of ripening, LPO inhibition in the seed extracts was 260% higher than in the peel-pulp extracts. In the seed extracts, LPO inhibitions in the unripe extracts were higher than those in the ripe extracts up to 84 µg/mL. However, the order of LPO inhibition changed inversely at higher concentrations. Among the peel-pulp extracts, the LPO inhibition rates were different between unripe and ripe papaya extracts after 430 µg/mL, remaining continuously higher than that of the unripe peel–pulp extracts at up to 3400 µg/mL. In the regression analysis, sigmoid curves were fitted only for all the seed extracts. The EC_50_ of ripe seed extracts (49.07 µg/mL) was significantly lower than that of unripe seed extracts (75.98 µg/mL).

FRAP was not detected for the unripe peel–pulp extracts but was detected in the other extracts (Figure 1D). The unripe seed extracts had the highest mean value (566.96 ± 6.57 µM FeSO_4_/g DW), followed by the ripe seed (217.18 ± 1.04 µM FeSO_4_/g DW) and ripe peel-pulp extracts (71.77 ± 1.10 µM FeSO_4_/g DW).

The evaluation of antioxidant activities has become a significant aspect on describing the nutritional and medicinal properties of food [38]. Currently, there is no standard method to determine the antioxidant activity of natural products. Various antioxidant activity assays can be employed to quantify antioxidant activities. In this study, DPPH and ABTS radical scavenging activities, LPO inhibition, and FRAP were compared to evaluate the antioxidant properties of papaya fruit parts during ripening. The tendencies of antioxidant activities were consistent among different assays, with the seed extracts having higher antioxidant activities than the peel-pulp extracts beyond a certain concentration, regardless of ripening.

Phenolics and flavonoids are major determinants of the antioxidant activities of papaya fruits [2]. In this study, total phenolic contents were strongly correlated with ABTS radical scavenging activity (r = 1.00) and FRAP (r = 1.00) (Table 3), whereas total flavonoid contents were not. Not all of the assays revealed an association between antioxidant activities and phenolic contents, as phenolic compounds preferably scavenge specific oxidizing agents. It should be noted that other bioactive compounds, including carotenoids and ascorbic acid, may contribute to the antioxidant activity of papaya fruits. Thus, comparative studies are essential for establishing specific antioxidant activities, as there is limited information available on papaya fruits [52]. Recently, Zhang et al. [38] reported that total phenolic content in different parts of the papaya fruit was correlated with DPPH and ABTS scavenging activity (r = 0.95), whereas total flavonoid content was correlated with FRAP (r = 0.92). As the antioxidant activity is driven by all contained phenolic compounds, ABTS and FRAP assays might allow for more accurate conclusions in this regard.

### 3.4. Anti-Inflammatory Activities

Cell viabilities were not significantly different from LPS-treated cells with the papaya extracts of 200 µg/mL as well as low concentrations (Figure S2) when compared to that of cells treated without papaya extracts; the papaya extracts were applied for determining ROS and NO productions, NF-κB activation, and iNOS and COX-2 expressions.

**Figure 2:**
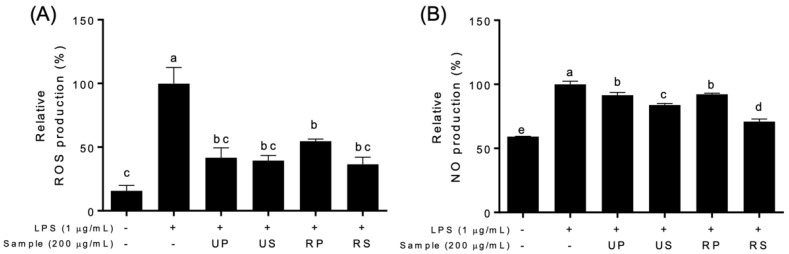
Effects of papaya extracts on the production of relative species in LPS-treated Raw-BLUETM cells; UP, unripe peel-pulp; US, unripe seed; RP, ripe peel-pulp; RS, ripening seed. (**A**) Relative oxygen species (ROS) production. (**B**) Nitric oxide (NO) production. Different letters indicate significant differences using the Tukey’s honestly significant difference test at *p* < 0.05. Vertical bars repre-sent standard errors of means. (n = 3).

LPS produced a significant amount of intracellular ROS and NO in RAW-Blue™ cells compared to control cells which were not treated with LPS and papaya extracts (Figure 2). All papaya extracts inhibited LPS-induced ROS and NO production to varying degrees among papaya extracts (Figure 2, Figures S3 and S4). The ROS productions were inhibited by all the papaya extracts; however, the inhibitory effects were not different among these extracts (Figure 2A). The ROS productions were inhibited to the low level of the control cells in all extracts except ripe peel–pulps. The NO productions were also inhibited at all papaya extracts (Figure 2B and Figure S4). Regardless of ripening, seed extracts had higher inhibitory effects than peel–pulp extracts. The inhibitory effects of seed extracts increased during ripening; those of peel–pulp extracts were maintained.

**Figure 3:**
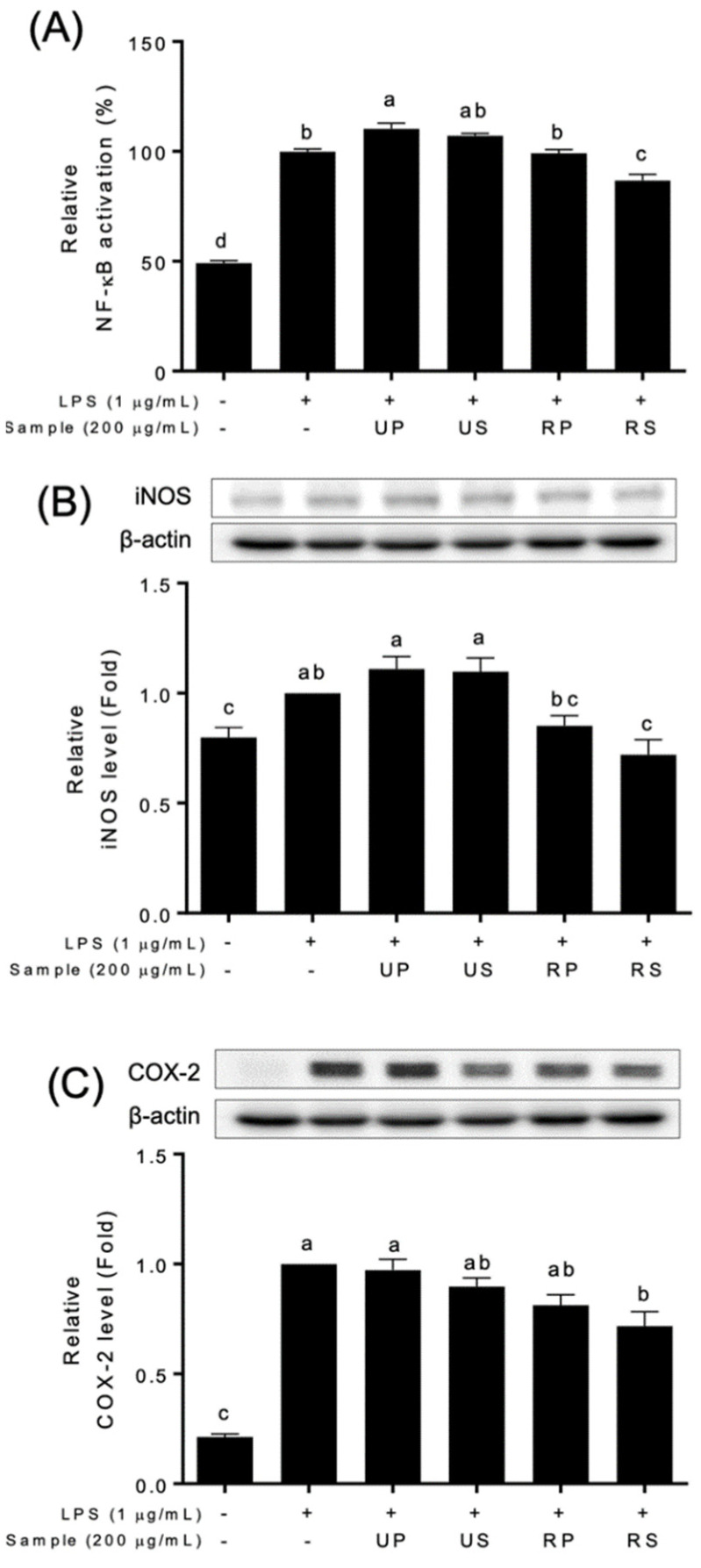
Effect of papaya fruit extracts on the inhibition of NF-κB signaling pathways in LPS-treated Raw-BLUETM cells; UP, unripe peel-pulp; US, unripe seed; RP, ripe peel-pulp; RS, ripening seed. (**A**) NF-κB activation; (**B**) iNOS levels; (**C**) COX-2 levels. Different letters indicate significant differences using the Tukey’s honestly significant difference test at *p* < 0.05. Vertical bars repre-sent standard errors of means. (n = 3).

Papaya extracts affected NF-κB signaling pathways (Figure 3 and Figure S5). The NF-κB activation were differently regulated among papaya extracts (Figure 3A and Figure S4). The unripe peel-pulp extracts upregulated, and the ripe seed extracts downregulated the NF-κB activation. The ripe peel-pulp and unripe seed extracts did not affect the activation. The expressions of NF-κB targets, iNOS and COX-2, were also different among papaya extracts (Figure 3B,C). These expressions decreased only in ripe seed extracts compared to the LPS-treated cells; those did not in other extracts.

The anti-inflammatory properties of papaya fruits have been recently reported using in vitro cell studies [19,39,53]. Previous studies reported that papaya extracts have an abilities to modulate inflammatory factors in various cell types exposed to different stresses [53]. The ripe seed extracts of ‘Caribbean red’ papaya fruits inhibited NO production in HepG2 cells [39]. The unripe fruit extracts of papaya fruits decreased the expression of iNOS and COX-2 in CRL-292^TM^ cells, although NO production was not inhibited [54]. In this study, ripe seed extracts showed a series of the inhibition of NO production (Figure 2), NF-κB activation, and iNOS and COX-2 productions (Figure 3). Pathak et al. [55] reported that the flavonoid-rich fraction of papaya seed extracts inhibited NF-κB activation as well as INFɣ, TNFɑ, and IL-6 in kidney, colon, lung, and pancreatic cells. Therefore, the inhibitory effects of ripe seed extracts (Figure 3) would be associated with flavonoid contents (Table 1). In addition, the contents of the identified flavonoid compounds (Table 2) were not consistent with the activities (Figure 2 and Figure 3). Further studies are needed to discover the effect of quantitative and qualitative effects of the compounds on anti-inflammatory activities.

## 4. Conclusions

Papaya fruit showed different antioxidant contents, and antioxidant and anti-inflammatory activities, depending on the ripening and fruit parts. Regardless of the ripening degree, the seed had higher phenolic contents than the peel-pulp, which was associated with antioxidant activity. Of individual phenolic compounds, each compounds had the different accumulation pattern as fruit parts and ripening. Seed extracts had higher antioxidant activity, especially, ABTS radical scavenging activity and FRAP accurately showed the relationship between total phenolics and the antioxidant activity. In anti-inflammatory activities, ripe seed extracts showed superior effects for the suppression of NF-κB signaling pathway. These results suggested papaya seed as well as peel–pulp has a potential for pharmacological raw materials. The data would provide fundamental data for the use of papayas for natural antioxidants.

## Figures and Tables

**Table 1:** Total phenol and flavonoid contents in papaya extracts.

Ripening Stage	Part	Total Phenolic Content (mg GAE/100 g DW)	Total Flavonoid Content (mg QUE/100 g DW)
Unripe	Peel-pulp	235 ± 0.3 ^d^	71.7 ± 0.06 ^a^
	Seed	2070 ± 1.3 ^a^	61.9 ± 0.05 ^a^
Ripe	Peel-pulp	568 ± 0.3 ^c^	67.8 ± 0.13 ^a^
	Seed	1080 ± 0.4 ^b^	117.7 ± 0.13 ^b^

Values are the mean with standard errors from three replicates with 12 fruit each. Means within columns followed by different superscript letters are significantly different at *p* < 0.05 using Tukey’s honest significant difference test.

**Table 2:** Relative amounts of individual phenolics. For the quantification of phenolics, relative retention times and relative peak areas were compared using fifteen compounds as standards. Of these compounds, five compounds were detected at least one extract: chlorogenic acid (CAS No. 327-97-9), cynarin (CAS No. 30964-13-7), eupatorine (CAS No. 855-96-9), neochlorogenic acid (CAS No. 906-33-2), and vicenin II (CAS No. 23666-13-9). The others were not detected in any papaya extract: acacetin (CAS 480-44-4), apigenin (CAS 520-36-5), apigetrin (CAS 528-74-5), cryptochlorogenic acid (CAS 905-99-7), diosmin (CAS 520-27-4), eridictol (CAS 20126-59-4), isoschaftoside (CAS 52012-29-0), linarin (CAS 480-36-4), luteolin (CAS 491-70-3), and luteoside (CAS 5373-11-5).

Ripening Stage	Part	Phenolic Acid			Flavonoid	
		Chlorogenic Acid	Cynarin	Neochlorogenic Acid	Eupatorine	Vicenin II
Unripe	Peel-pulp	0.22 ± 0.15	10.21 ± 0.61 ^a^	2.02 ± 0.21 ^b^	-	-
	Seed	-	-	8.73 ± 0.16 ^a^	4.70 ± 0.43 ^a^	-
Ripe	Peel-pulp	-	5.30 ± 0.13 ^b^	2.98 ± 0.23 ^b^	-	2.90 ± 0.13 ^a^
	Seed	-	-	0.90 ± 0.06 ^c^	2.02 ± 0.11 ^b^	1.06 ± 0.09 ^b^

Values are the mean with standard errors from three replicates with 12 fruit each. Means within columns followed by different superscript letters are significantly different at *p* < 0.05 using Tukey’s honest significant difference test; -, not detected.

**Table 3:** Correlation coefficients for the relationships between total phenol content, total flavonoid content, 2,2′-diphenyl-1-picrylhydrazyl (DPPH) radical scavenging activity, 2,2′-azino-bis-(3-ethylbenzothiazoline-6-sulfonate) (ABTS) radical scavenging activity, inhibition of lipid peroxidation (LPO), and ferric reducing antioxidant power (FRAP).

Antioxidant	DPPH	ABTS	LPO	FRAP
Total phenols	−0.64	−1.00 *	−0.77	1.00 *
Total flavonoids	0.92	0.20	−0.40	−0.32

* Significant at *p* < 0.05.

## Data Availability

Not applicable.

## Supplementary Materials

The following supporting information can be downloaded at: https://www.mdpi.com/article/10.3390/foods11203211/s1, Figure S1: Representative HPLC profiles of (up) phenolic compounds and (down) papaya extracts. 1, neochlorogenic acid; 2, cryptochlorogenic acid; 4, eupatorine; 5, vicenin II; 6, isoschaftoside; 7, cynarin; 8, isochlorogenic acid; 9, isochlorogenic acid; 10, apigetrin; 11, isochlorogenic acid; 12, eridictiol; 13, linarin; 14, luteolin; 15, apigenin; 16, diosmein; 17, eupatorine; 18, acacetin; Figure S2: Effects of papaya fruit extracts of four concentrations (0, 60, 80, and 200 µg/mL) on cell viabilities in LPS-treated Raw-BLUETM cells. (A) Unripe peel-pulp (UP) extracts. (B) unripe seed (US) extracts. (C) ripe peel-pulp (RP) extracts. (D) ripe seed (RS) extracts. N.S. indicates no-significant differences using one-way ANOVA at *p* < 0.05. Vertical bars represent standard errors of means. (n = 3); Figure S3: The fluorescence images of ROS in papaya fruit extracts at 200 µg/mL in LPS-treated Raw-BLUE^TM^ cells; UP, unripe peel-pulp extracts; US, unripe seed extracts; RP, ripe peel-pulp extracts; RS, ripe seed extracts; Figure S4: Effects of papaya fruit extracts of four concentrations (0, 60, 80, and 200 µg/mL) on NO production in LPS-treated Raw-BLUE^TM^ cells. (A) Unripe peel-pulp (UP) extracts. (B) unripe seed (US) extracts. (C) ripe peel-pulp (RP) extracts. (D) ripe seed (RS) extracts. Different letters indicate significant differences using the Tukey’s honestly significant difference test at p < 0.05. Vertical bars represent standard errors of means. (n = 3); Figure S5: Effects of papaya fruit extracts of four concentrations (0, 60, 80, and 200 µg/mL) on NF-κB activation in LPS-treated Raw-BLUE^TM^ cells. (A) Unripe peel-pulp (UP) extracts. (B) unripe seed (US) extracts. (C) ripe peel-pulp (RP) extracts. (D) ripe seed (RS) extracts. Different letters indicate significant differences using the Tukey’s honestly significant difference test at *p* < 0.05. Vertical bars represent standard errors of means. (n = 3).
