# Supplementary material for: Comprehensive Assessment of Antioxidant and Anti-Inflammatory Properties of Papaya Extracts
Source: Foods. 2022 Oct 14;11(20):3211. doi: 10.3390/foods11203211 (PMC9601897; doi:10.3390/foods11203211)
Supplement: Supplementary file 1 [file foods-11-03211-s001.zip › foods-1922836-supplementary.pdf]

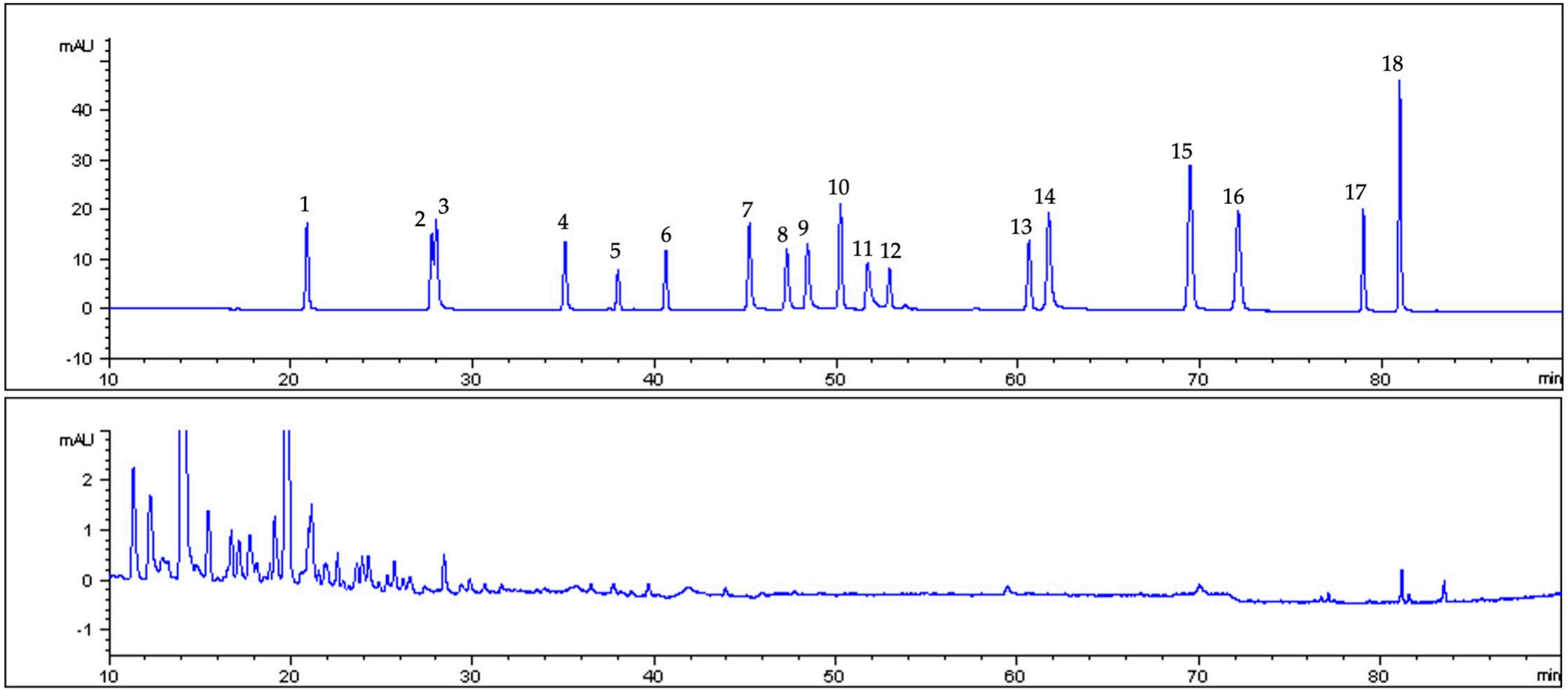

Figure S1. Representative HPLC profiles of (up) phenolic compounds and (down) papaya extracts. 1, neochlorogenic acid; 2, cryptochlorogenic acid; 4, eupatorine; 5, vicenin II; 6, isoschaftoside; 7, cynarin; 8, isochlorogenic acid; 9, isochlorogenic acid; 10, apigetrin; 11, isochlorogenic acid; 12, eridictiol; 13, linarin; 14, luteolin; 15, apigenin; 16, diosmein; 17, eupatorine; 18, acacetin.

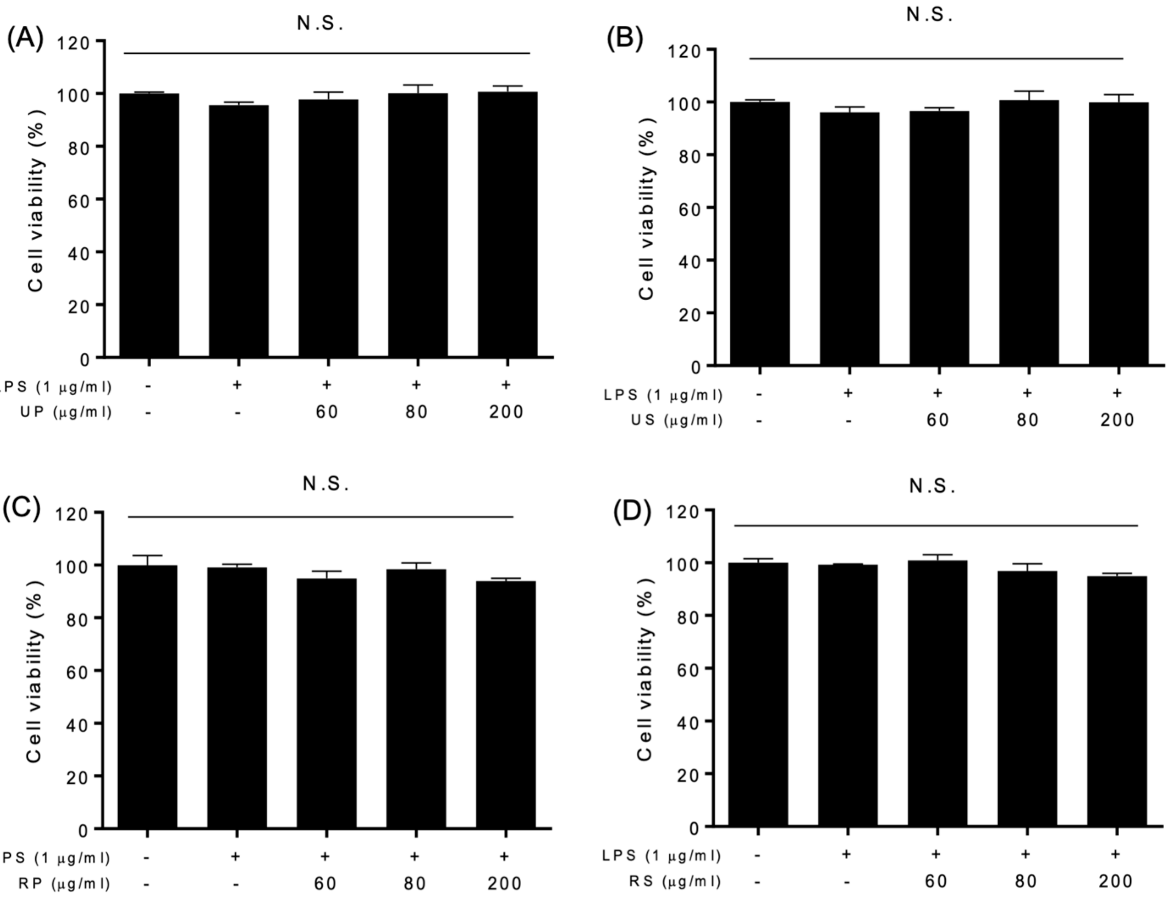

Figure S2. Effects of papaya fruit extracts of four concentrations (0, 60, 80, and 200 μg/mL) on cell viabilities in LPS-treated Raw-BLUE^{TM} cells. (**A**) Unripe peel-pulp (UP) extracts. (**B**) unripe seed (US) extracts. (**C**) ripe peel-pulp (RP) extracts. (**D**) ripe seed (RS) extracts. N.S. indicates no-significant differences using one-way ANOVA at $p < 0.05$. Vertical bars represent standard errors of means. (n = 3).

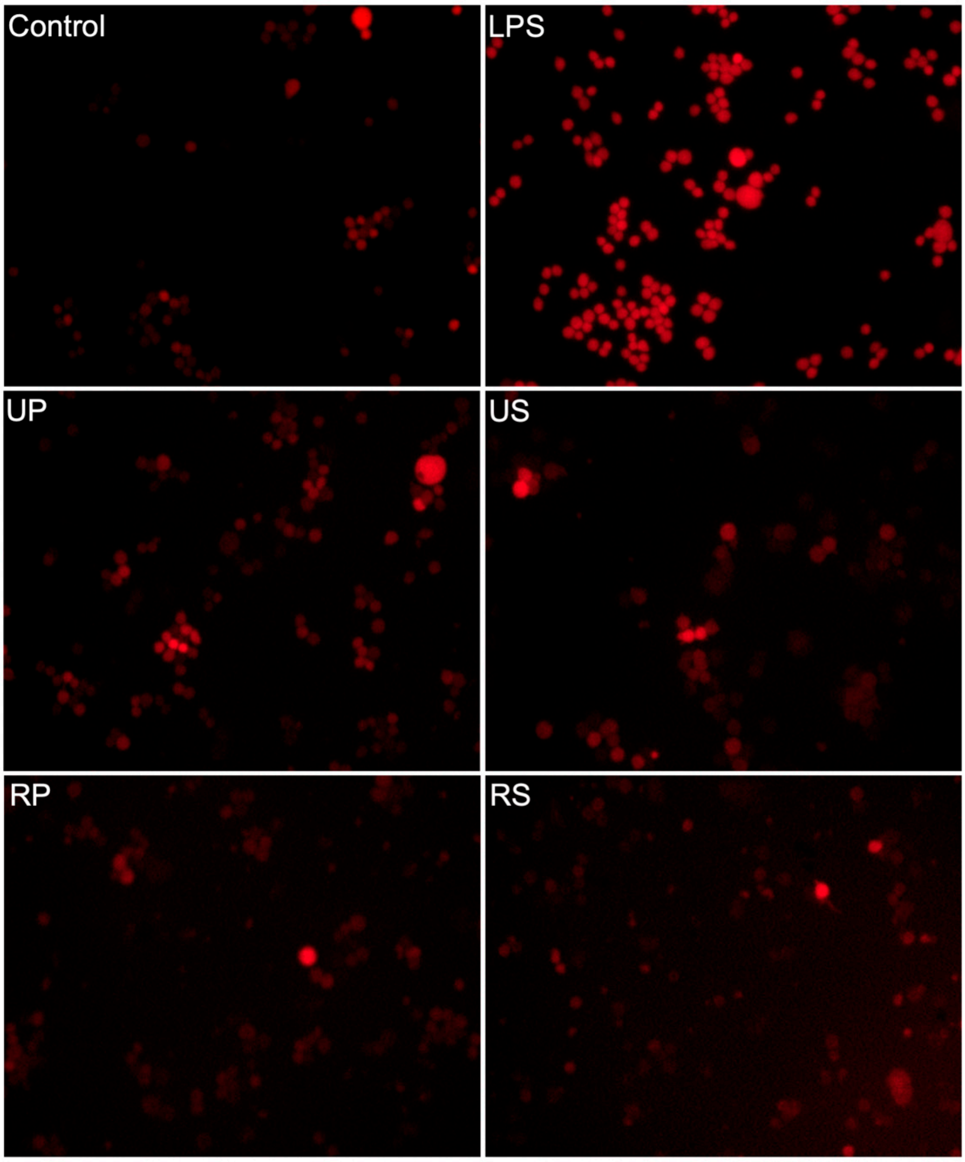

Figure S3. The fluorescence images of ROS in papaya fruit extracts at 200 μg/mL in LPS-treated Raw-BLUE™ cells; UP, unripe peel-pulp extracts; US, unripe seed extracts; RP, ripe peel-pulp extracts; RS, ripe seed extracts.

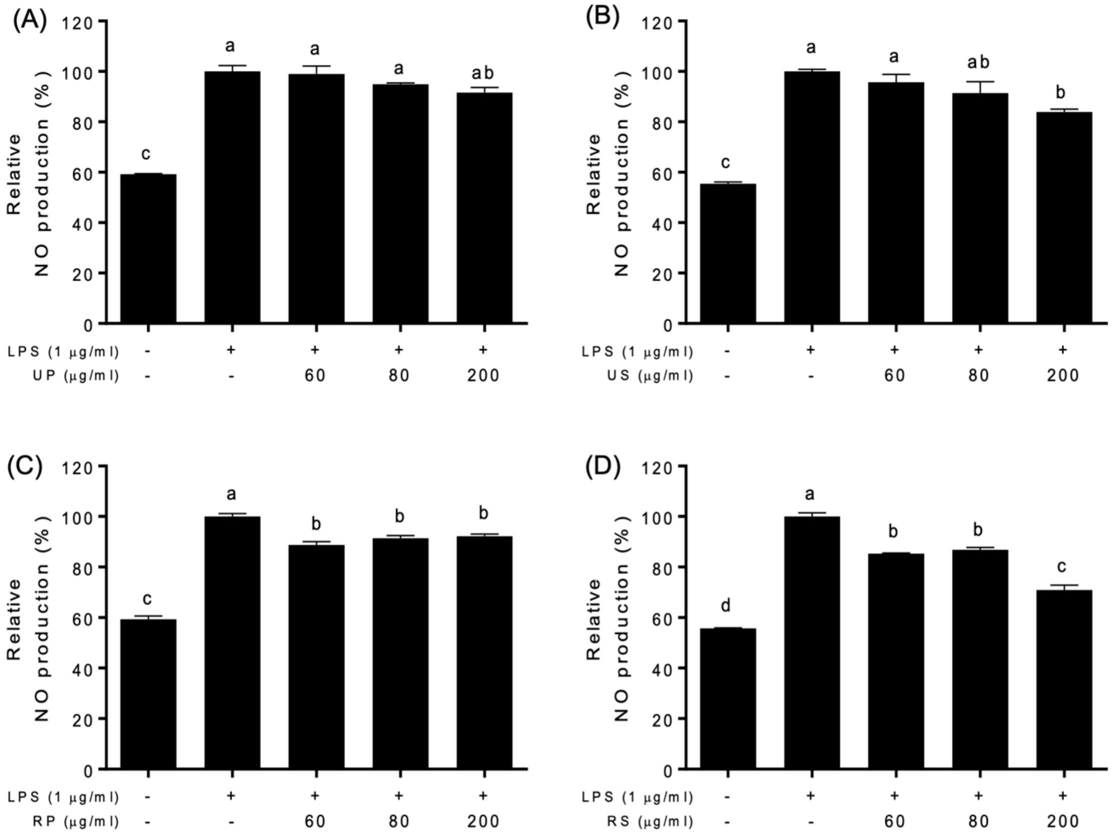

Figure S4. Effects of papaya fruit extracts of four concentrations (0, 60, 80, and 200 μg/mL) on NO production in LPS-treated Raw-BLUE™ cells. (**A**) Unripe peel-pulp (UP) extracts. (**B**) unripe seed (US) extracts. (**C**) ripe peel-pulp (RP) extracts. (**D**) ripe seed (RS) extracts. Different letters indicate significant differences using the Tukey's honestly significant difference test at $p < 0.05$. Vertical bars represent standard errors of means. (n = 3).

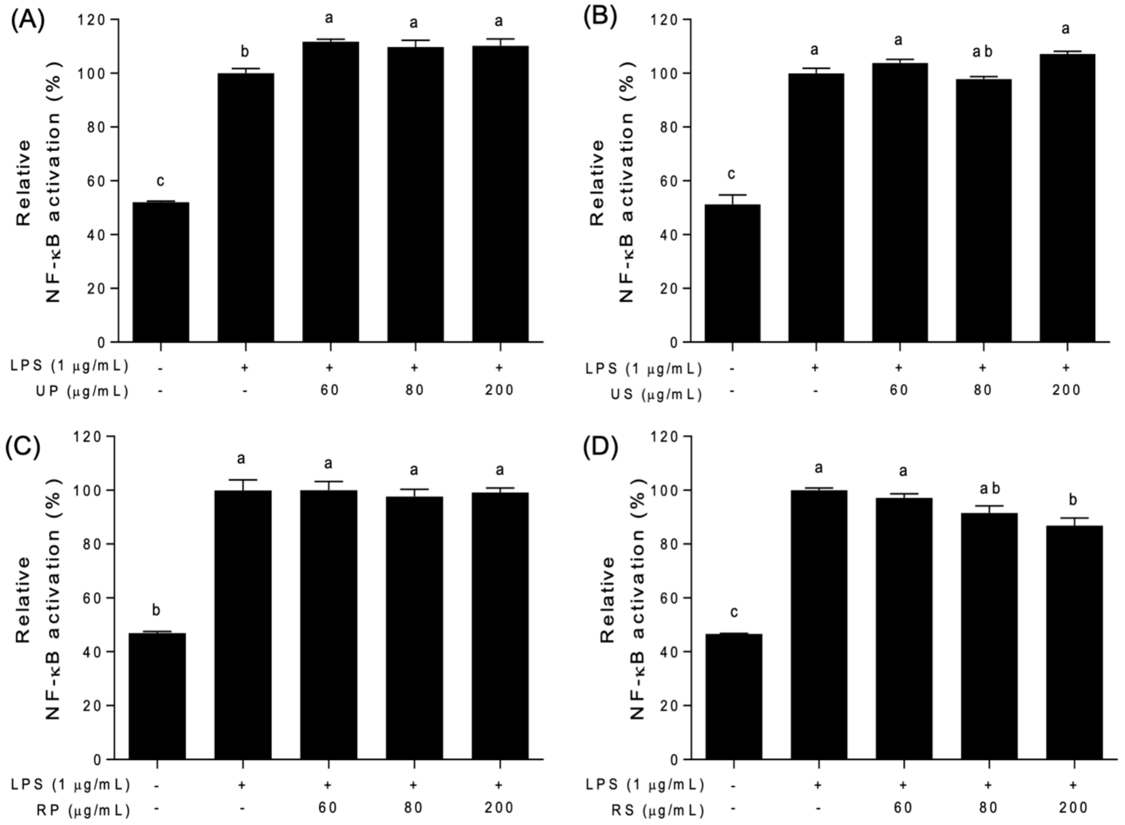

Figure S5. Effects of papaya fruit extracts of four concentrations (0, 60, 80, and 200 µg/mL) on NF-κB activation in LPS-treated Raw-BLUE™ cells. (**A**) Unripe peel-pulp (UP) extracts. (**B**) unripe seed (US) extracts. (**C**) ripe peel-pulp (RP) extracts. (**D**) ripe seed (RS) extracts. Different letters indicate significant differences using the Tukey's honestly significant difference test at $p < 0.05$. Vertical bars represent standard errors of means. (n = 3).
